# Serum Levels of IL-1***β***, IL-6, TGF-***β***, and MMP-9 in Patients Undergoing Carotid Artery Stenting and Regulation of MMP-9 in a New *In Vitro* Model of THP-1 Cells Activated by Stenting

**DOI:** 10.1155/2015/956082

**Published:** 2015-05-31

**Authors:** Rongrong Zhang, Fan Jiang, Cindy Si Chen, Tianzhu Wang, Jinzhou Feng, Tao Tao, Xinyue Qin

**Affiliations:** ^1^Department of Neurology, The First Affiliated Hospital of Chongqing Medical University, Chongqing 400016, China; ^2^Department of Neurology, The Fifth People's Hospital of Chongqing, Chongqing 400062, China; ^3^Department of Medicine, Drexel University College of Medicine, Philadelphia, PA 19129, USA; ^4^Department of Neurology, Affiliated Hospital of Luzhou Medical College, Luzhou 646000, China

## Abstract

Inflammation plays an important role in the pathophysiological process after carotid artery stenting (CAS). Monocyte is a significant source of inflammatory cytokines in vascular remodeling. Telmisartan could reduce inflammation. In our study, we first found that, after CAS, the serum IL-1*β*, IL-6, TGF-*β*, and MMP-9 levels were significantly increased, but only MMP-9 level was elevated no less than 3 months. Second, we established a new *in vitro* model, where THP-1 monocytes were treated with the supernatants of human umbilical vein endothelial cells (HUVECs) that were scratched by pipette tips, which mimics monocytes activated by mechanical injury of stenting. The treatment enhanced THP-1 cell adhesion, migration and invasion ability, and the phosphorylation of ERK1/2 and Elk-1 and MMP-9 expression were significantly increased. THP-1 cells pretreated with PD98095 (ERK1/2 inhibitor) attenuated the phosphorylation of ERK1/2 and Elk-1 and upregulation of MMP-9, while pretreatment with telmisartan merely decreased the phosphorylation of Elk-1 and MMP-9 expression. These results suggested that IL-1*β*, IL-6, TGF-*β*, and MMP-9 participate in the pathophysiological process after CAS. Our new *in vitro* model mimics monocytes activated by stenting. MMP-9 expression could be regulated through ERK1/2/Elk-1 pathway, and the protective effects of telmisartan after stenting are partly attributed to its MMP-9 inhibition effects via suppression of Elk-1.

## 1. Introduction

Cerebral ischemia is one of the leading causes of death in the world. Carotid artery stenosis is one of the main risk factors of the development of ischemic stroke, representing approximately 20% of the total incidence [1]. Preventing carotid artery stenosis is a major target in averting primary and secondary stroke. Carotid artery stenting (CAS), a less invasive alternative technique to carotid endarterectomy, has become one of the major treatment modalities for carotid artery stenosis in recent years. Although stents are successful in the majority of cases, many patients still suffer serious complications of stenting such as in-stent restenosis (ISR), which is a main concern in clinical practice.

Inflammation has been widely demonstrated to play a central role in the pathophysiological process of restenosis after CAS by causing neointimal hyperplasia [2, 3]. After stenting, endothelial disruption and abrasion are induced by the infiltration of the balloon and implantation of the stent. This mechanical injury triggers substantial local inflammation, stimulates vascular smooth muscle cell proliferation and extracellular matrix deposition, and leads to neointimal thickening and restenosis. This inflammatory process involves the production of multiple pro- and anti-inflammatory factors that are released by complex interactions between multiple cell types after CAS [3–5]. In coronary artery stenting, proinflammatory factors interleukin-1*β* (IL-1*β*), interleukin-6 (IL-6) and anti-inflammatory factor transforming growth factor-*β* (TGF-*β*) play important roles in the early inflammatory process and the development of ISR after stenting, and elevation of matrix metalloproteinase-9 (MMP-9) continues for a long time and is associated with coronary artery in-stent restenosis [6–11]. To date, it is still unclear whether those factors also take part in the pathophysiological process after CAS and whether they take part in both the acute and chronic phase.

MMP-9, a member of matrix metalloproteinases family, works by degrading various components of the extracellular matrix and changing nonextracellular matrix molecule expression and may play a pivotal role in the pathogenesis of restenosis after stenting [6, 8, 9, 12]. MMP-9 is produced by various cells, including monocytes [13, 14]. Monocyte is a significant source of MMP-9 and plays an important role in vascular remodeling in atherosclerosis [13]. After stenting, endothelial injury or endothelial stress would lead to the infiltration of circulating monocytes in stented arteries [15–17]. Then, facilitated by the presence of cellular adhesion molecules, such as vascular cell adhesion molecule-1 (VCAM-1), the monocytes adhere to endothelial cells and subsequently migrate into the intima. After migration, the monocytes differentiate into macrophages or dendritic cells, which contribute to ISR and stent thrombosis. Monocytes could be activated by soluble factors secreted by the injured endothelial cells. During this process, expression of MMP-9 in monocytes could be induced [15, 18–20]. But to our knowledge, there is no* in vitro* model which mimics the activation of monocytes stimulated by excretion of stent-induced injured endothelial cells.

Expression of MMP-9 has been widely confirmed to be regulated by the activation of extracellular signal-regulated kinases 1/2 (ERK1/2) in various pathologic conditions [21, 22]. Elk-1, a member of the ternary complex factor subfamily of Ets (E-twenty six) domain transcription factors, is well known to be phosphorylated by ERK1/2, which transforms Elk-1 from a transcriptionally repressive to a transcriptionally active form [23]. Moreover, the promoter of MMP-9 has been shown to possess a functional enhancer element-binding site for Elk-1, and several reports indicate that MMP-9 expression could be regulated by Elk-1 in the ERK1/2 signal pathway [24–26]. However, the mechanism of the ERK1/2/Elk-1 pathway mediated MMP-9 upregulation in the monocytes stimulated by mechanical injury of stenting has not yet been clarified.

Telmisartan is a unique angiotensin II receptor blocker (ARB) and a partial agonist of peroxisome proliferator-activated receptor-gamma (PPAR-*γ*). Recent clinical trials found that telmisartan reduced inflammation, late lumen loss, and neointimal tissue proliferation in patients who underwent coronary artery stenting [27–29]. The inhibitory effects of telmisartan on MMP-9 expression are also well documented [30–34]. Additionally, PPAR-*γ* agonists are reported to suppress MMP-9 expression by blocking activator protein-1 (AP-1) activity, which could be regulated by ERK1/2/Elk-1 pathway [35–37]. Thus, as a partial PPAR-*γ* agonist, telmisartan may inhibit increased MMP-9 expression through the ERK1/2/Elk-1 pathway. There have been no previous studies on the effect of telmisartan on MMP-9 expression and this mechanism in monocytes following stenting.

Based on these observations, we first monitored the serial serum levels of inflammatory cytokines IL-1*β*, IL-6, TGF-*β*, and MMP-9 in patients who underwent CAS with bare metal stents at 6-month follow-up to study the roles of these factors after stenting. The results indicate that MMP-9 is involved in the pathophysiological process for a long time after stenting. We set up a novel* in vitro* model of THP-1 monocytes activated by the supernatants of scratch-injured endothelial cells to mimic the activation of monocytes after CAS. We use this new model to investigate the involvement of the ERK1/2/Elk-1 pathway in MMP-9 expression in monocytes after stenting. Lastly, we determined if telmisartan suppresses the expression of MMP-9 in this model through an ERK/Elk-1-mediated pathway.

## 2. Materials and Methods

### 2.1. Ethics Statement

This study was approved by the Institutional Ethics Committee of the First Affiliated Hospital of Chongqing Medical University, and written informed consent was obtained from all potential study candidates before any procedure.

### 2.2. Patients

From September 2009 through December 2011, a prospective cohort of patients who were scheduled for CAS was recruited from the First Affiliated Hospital of Chongqing Medical University, China. Patients were eligible for CAS if they were suffering from carotid atherosclerosis (stenosis degree ≥50% in symptomatic patients or ≥70% in asymptomatic patients) confirmed by computer tomography angiography (CTA) or digital subtraction angiography (DSA). Symptomatic patients had to have a history of at least one ipsilateral ischemic event within the last 6 months, while asymptomatic patients did not have neurologic symptoms. Patients with the following conditions were excluded from the study: stenosis from causes other than atherosclerosis, restenosis, concurrent severe illness (such as neoplastic disease, hepatic or renal disease, or infection), a potential cause for neurological symptoms other than atherosclerotic carotid stenosis, a recent stroke or acute coronary syndrome during the preceding 2 weeks, alcohol abuse, steroid therapy, or an injury or surgical procedure in the period of 6 months before assaying the serum levels of the inflammatory cytokines.

Baseline demographic and clinical information was obtained from patient interview, physical imaging, and laboratory examination. The information included age, sex, distributions of classic cerebrovascular disease risk factors (hypertension, coronary artery disease, diabetes, impaired glucose tolerance, hyperlipidemia, and hyperthyroidism), symptomatic/asymptomatic, and degree of stenosis.

### 2.3. CAS Treatment Process, Clinical Follow-Up, and Serum Sample Collection

CAS was performed by experienced interventional neuroradiologists and done under anesthesiological standby. The vascular system was accessed via the femoral artery. CAS was performed using a bare metal stent. Stent type and the use of filter-based neuroprotective devices were chosen at the discretion of the interventionalists. Aspirin and clopidogrel were started for at least 3 days before the procedure and were continued for at least 1 month after the procedure. Then, the patients would be given aspirin or clopidogrel indefinitely. Complete neurologic examinations were performed by an independent neurologist before, 24 hours after, and 3 months and 6 months after CAS. Medication use, complications, cerebrovascular events, carotid ultrasound or CTA to detect carotid artery ISR, and blood samples to identify inflammatory biomarkers were evaluated prior to stenting, at 24 hours, 3 and 6 months after CAS procedure. Venous blood was taken in the morning from the antecubital vein with minimal stasis, placed in a coagulation promoting tube and stored at 4°C for 1 hour. Then the blood was centrifuged at a speed of 3000 rpm for 10 min and the serum samples were immediately stored at −80°C before being analyzed.

### 2.4. Cell Line and Cell Culture

Human umbilical vein endothelial cells (HUVECs) and THP-1 cells, a human monocytic leukemia cell line, were both obtained from the Shanghai Institute of Biochemistry and Cell Biology, the Chinese Academy of Sciences (Shanghai, China). HUVECs were grown in DMEM medium (Gibco, Calif, USA) supplemented with 10% fetal bovine serum (FBS) (Gibco, Calif, USA). THP-1 cells were cultured in RMPI 1640 medium (Gibco, Calif, USA) containing 10% FBS. All cells were maintained at 37°C in a humidified incubator containing 5% CO_2_ and the medium was changed every 2-3 days. In some experiments, THP-1 cells were pretreated with 0.02 mg/*μ*L ERK1/2 inhibitor PD98095 (Beyotime, Shanghai, China) or 10 *μ*mol/L telmisartan (Sigma, MO, USA).

### 2.5. Mechanical Injury and Adhesion Assay

HUVECs (5.0 × 10^4^ cells/mL) were grown in 24-well plates for 12 hours. Scratch wounds (twice, 5 times, or 10 times) were made across the cell layer using 200 *µ*L pipette tips. Cultures were scratched by a pipette tip twice as the minor injury group, five times as the mild injury group, and ten times as the severe injury group. The control group was cultured without any scratch injury. Afterwards, the plates were washed twice with phosphate-buffered saline (PBS) and then incubated at 37°C in RPMI 1640 without supplemented FBS for 24 hours. Next, the supernatants of the endothelial cells of each group were collected.

HUVECs were cultured in 96-well plates for 12 hours and divided into 4 different groups. Culture media in each group were replaced by the different collected supernatants according to their grouping. Then the HUVECs were incubated with 2 × 10^4^ THP-1 cells. After incubation for 1 hour, plates were washed by PBS and then RPMI 1640 medium was added without FBS. Afterwards, MTT (Sigma, MO, USA) was applied to measure adhesion ability. The results were expressed as adhesion rates. According to our results, the five times of scratch would lead to highest adhesion rates. So we chose the supernatants of HUVECs which were scratched five times as the supernatants of the injured group in the following study.

### 2.6. Chemotaxis and Invasion Assay

To observe the ability of the supernatants of scratch-injured HUVECs on THP-1 cell migration and invasion, chemotaxis and invasion assays were performed in 8 *µ*m transwell plates (Millipore, MA, USA).

In the chemotaxis assay, 5 × 10^5^ THP-1 cells in 100 *µ*L volume of culture were added to the upper chamber. In the invasion assay, 40 *µ*L of diluted Matrigel was put into the upper chamber of a 24-well transwell and incubated at 37°C for 1 hour. Then, 1 × 10^6^ THP-1 cells in 100 *µ*L volume of culture were added to the upper chamber. Afterwards, the supernatants were added to the lower chamber of the transwell for both chemotaxis and invasion assays. After 24 hours of incubation, cells were washed with PBS and fixed with paraformaldehyde. Cells were then stained with Giemsa (Sigma, MO, USA) for 5 minutes. The results of the chemotaxis and invasion assays were evaluated by counting the number of migrating or invading cells under a microscope.

### 2.7. ELISA

Serum levels of IL-1*β*, IL-6, TGF-*β*, and MMP-9 and the concentrations of monocyte chemoattractant protein-1 (MCP-1) and soluble vascular cell adhesion molecule-1 (sVCAM-1) in the supernatants of the injury and control group were measured by enzyme-linked immunosorbent assay (ELISA), using commercially available high-sensitivity ELISA kits (Boster, Wuhan, China) according to the manufacturers' instructions.

### 2.8. Cell Proliferation Assay

THP-1 cells were cultured in 96-well plates at 5 × 10^3^ cells/well and incubated at 37°C in an incubator containing 5% CO_2_ for 12 hours. Then different concentrations of telmisartan (1.25, 2.5, 5, 10, 20, and 40 *µ*mol/L) were added to each well, respectively, and then the cells were incubated for 24, 48, or 72 hours. Cell and blank control group were set as well. Afterwards, 20 *µ*L of MTT was added to each well and incubated for 4 hours. Then the plates were centrifuged at a speed of 2000 rpm for 10 minutes. After removing the supernatants, 200 *µ*L of dimethylsulfoxide (DMSO) was added to each well. After shaking the plate for 15 min in the shaking board, the absorbance of each well was measured. The cell inhibitory rate was calculated according to the following formula: inhibitory rate = [(the absorbance of cell control group − the absorbance of blank control group)/(the absorbance of experimental groups − the absorbance of blank control group)] × 100%.

### 2.9. Western Blot

Western blot was performed to examine the phosphorylation of ERK1/2 and Elk-1 and MMP-9 expression in THP-1 cells. Cells were washed twice with ice-cold PBS and lysed in lysis buffer containing phenylmethylsulfonyl fluoride (PMSF), proteinase inhibitor, and phosphatase inhibitor. These lysates were incubated on a rocking platform for 15 minutes at 4°C and centrifuged at 12,000 rpm for 15 minutes at 4°C. The protein was extracted using a protein extraction kit (KeyGEN Biotech, Nanjing, China), according to the manufacturer's instructions. Protein concentration was determined using a BCA protein assay kit (KeyGEN Biotech, China). Proteins were separated in SDS-PAGE gels (10%) and transferred to PVDF membranes (Millipore, MA, USA). After blocking with 5% nonfat milk for 1 h, membranes were incubated with the following primary antibodies: p-ERK1/2 (1 : 2000 dilution, Cell Signaling Technology, MA, USA), p-Elk-1 (1 : 400 dilution, Santa Cruz Biotechnology, CA, USA), MMP-9 (1 : 100 dilution, Santa Cruz Biotechnology, CA, USA), and GAPDH (1 : 2000 dilution, Good Here, Hangzhou, China) at 4°C overnight. After washing in PBST three times, membranes were incubated with appropriate HRP-conjugated secondary antibody (1 : 3000 dilution, ZSGB-Bio, China) at 37°C for 1 hour. Antibodies were detected using ECL reagent kits (KeyGEN Biotech, Nanjing, China) and exposed to the Chemi Doc XRS imaging system (Bio-Rad, USA). The density of the expressed bands was quantified by densitometry with Quantity One software (Bio-Rad Laboratories).

### 2.10. Statistical Analysis

All results are presented as mean ± standard deviation (mean ± SD). Statistical differences among multiple groups were performed using one-way analysis of variance (ANOVA) followed by a post hoc LSD test. Unpaired Student's *t*-test was used for comparison between two groups when appropriate. Values of *P* < 0.05 were considered statistically significant. All data was carried out using SPSS 17.0 software.

## 3. Results

### 3.1. Clinical Study

#### 3.1.1. Baseline Characteristics and Clinical Follow-Up

We enrolled 74 patients scheduled for CAS. The baseline characteristics are shown in Table 1. Successful stent implantation was achieved in all patients (100%), and all of them were able to be accessed during the follow-up. Three patients (4.05%) showed a bleeding tendency 24 hours after CAS and we did not find any other complications in the rest of the cases. 6.76% of cases (*n* = 5) presented with dizziness 24 hours after CAS procedure. At the 6-month follow-up, total adverse cerebrovascular events included 1 death (1.35%), 1 transient ischemic attack (1.35%), and 2 strokes (2.70%). We did not find any evidence of restenosis in all cases during the follow-up time. Medical treatment after CAS included the use of antiplatelet agents in all patients (100%), antihyperlipidemics, hypoglycemic drugs, antihypertensive agents, and antithyroid drugs if needed.

#### 3.1.2. Serum Levels of IL-1*β*, IL-6, TGF-*β*, and MMP-9

**Figure 1:**
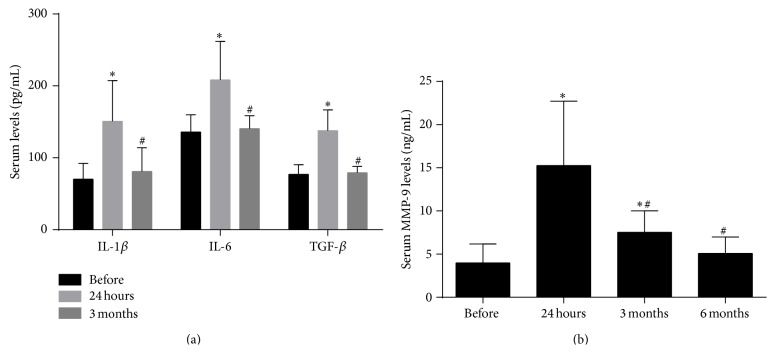
Interleukin-1*β* (IL-1*β*), interleukin-6 (IL-6), transforming growth factor-*β* (TGF-*β*), and matrix metalloproteinase-9 (MMP-9) serum levels at different time intervals. (a) Serum levels of IL-1*β*, IL-6 and TGF-*β* before, 24 hours and 3 months after carotid artery stenting (CAS). (b) Serum MMP-9 levels before, 24 hours, 3 months, and 6 months after CAS. ^*∗*^ *P* < 0.05 compared with before; ^#^ *P* < 0.05 compared with 24 hours.

We analyzed the serum levels of IL-1*β*, IL-6, TGF-*β*, and MMP-9 before stenting, at 24 hours, 3 and 6 months after the CAS procedure to determine whether they are involved in the pathophysiological process after CAS. Concentrations of IL-1*β*, IL-6, and TGF-*β* were significantly elevated 24 hours after CAS and returned to the preoperative levels less than 3 months after the operation (Figure 1(a)). MMP-9 levels also increased 24 hours after stenting, but the high expression continued for at least 3 months after stent implantation (Figure 1(b)). This indicates that MMP-9, a molecule widely reported to be associated with the process of ISR in coronary artery stenting, also takes part in the long-term pathophysiological process after CAS.

### 3.2. *In Vitro* Study

#### 3.2.1. Supernatants of Scratch-Injured HUVECs Induce Activation of THP-1 Cells

**Figure 2:**
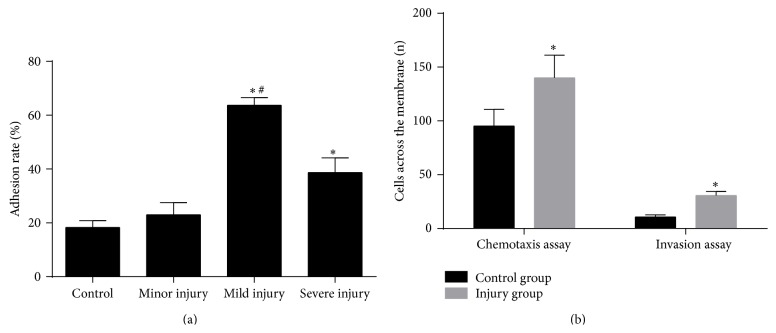
Scratch-injured human umbilical vein endothelial cells (HUVECs) supernatants induce THP-1 cells activation. (a) Results of the adhesion assay. (b) Results of the chemotaxis and invasion assays. ^*∗*^ *P* < 0.05 compared with control group; ^#^ *P* < 0.05 compared with severe injury group.

To study the regulation of MMP-9 in monocytes after stenting, we developed a new* in vitro* model of THP-1 cells incubated with the supernatants of scratch-injured HUVECs. Scratch injury mimics the mechanical injury induced by stenting. This new model intends to mimic monocytes activated by soluble factors released by mechanically injured endothelia cells. In order to evaluate the effect of the new model, we first examined the adhesive capacity of the THP-1 cells to HUVECs, which is critical in the inflammatory process after stenting [3]. Incubation with the supernatants of injured HUVECs increased the adhesion of THP-1 cells to HUVECs (Figure 2(a)). In the mild and severe injury group, the adhesion rates were significantly higher compared to the control group, while there was no significant difference between the minor injury group and the control group. Furthermore, the supernatants of the mild injury group increased the adhesion rate more than that of the severe injury group. These results suggest that the supernatants of cultures which were scratched five times (mild injury group) will be the most suitable in the process of establishing our new model as it promotes adhesion most clearly. To further confirm the activation of THP-1 cells stimulated by the injured endothelial cell supernatants, we measured the migration and invasion ability of these cells by using chemotaxis and invasion assays.  Figure 2(b) shows that, in comparison with the control group, the number of THP-1 cells that passed through the membrane of the transwell in the chemotaxis and invasion assays were both clearly increased in injured groups. Taken together, our results showed that stimulation of scratch-injured HUVEC supernatants induces THP-1 cells activation, as it promotes THP-1 cell adhesion to HUVECs and enhances THP-1 cell migration and invasion ability.

#### 3.2.2. Effects of Scratch Injury on MCP-1 and sVCAM-1 Concentrations in HUVECs Supernatants

**Figure 3:**
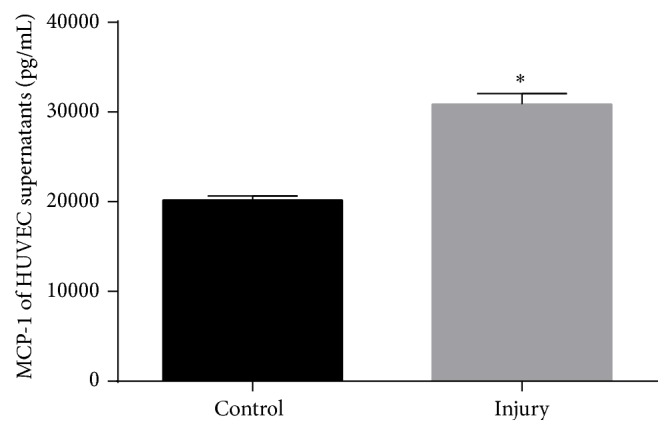
Effects of scratch injury on monocyte chemoattractant protein-1 (MCP-1) concentrations in human umbilical vein endothelial cells (HUVECs) supernatants. ^*∗*^ *P* < 0.05 compared with control.

Previous study suggests that blood concentrations of MCP-1 and sVCAM-1 in patients who underwent stent implantation were elevated after stenting [38–40]. Both of them play important roles in monocytes activation [41–44]. Hence, the effects of scratch injury on MCP-1 and VCAM-1 excretion by endothelia cells were studied. In supernatants of scratch-injured HUVECs, a significant increase expression of MCP-1 was observed (Figure 3), while there is no significant change in sVCAM-1 concentration (data not shown).

#### 3.2.3. ERK1/2/Elk-1-Mediated MMP-9 Expression in THP-1 Cells Induced by Scratch-Injured HUVECs Supernatants

**Figure 4:**
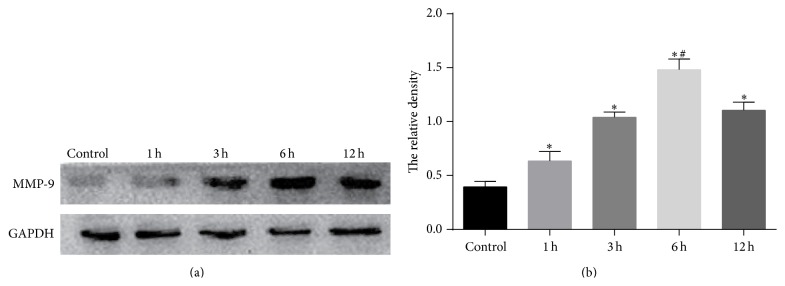
Scratch-injured human umbilical vein endothelial cells (HUVECs) supernatants induced MMP-9 expression in THP-1 cells. (a) MMP-9 protein expression in control cultures and in cultures treated with the supernatants of scratch-injured HUVECs for 1, 3, 6, and 12 h. (b) Quantitative data for (a). ^*∗*^ *P* < 0.05 compared with control; ^#^ *P* < 0.05 compared with rest groups.

Our results suggested that serum MMP-9 remained at an elevated level for at least 3 months after CAS, and MMP-9 expression is well known to be associated with clinical ISR after coronary stenting [6, 8, 9]. We then explored the mechanism of MMP-9 expression in monocytes after stenting using our new model. First, we performed Western blot to find the expression of MMP-9 in THP-1 cells. The results revealed that injured endothelial supernatants stimulate a time-dependent increased expression of MMP-9 with a maximum response at 6 hours after stimulation (Figures 4(a) and 4(b)).

**Figure 5:**
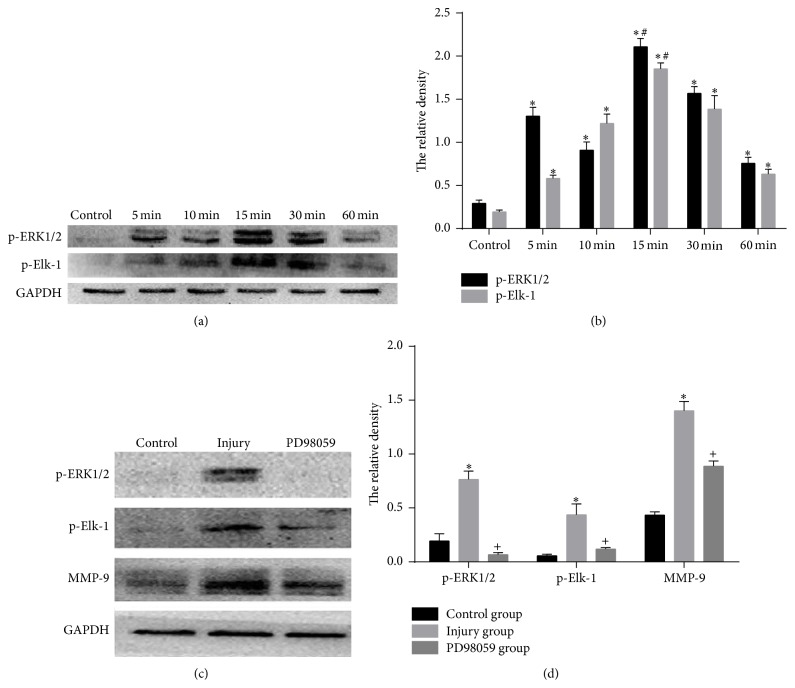
MMP-9 expression is modulated by the ERK1/2/Elk-1 pathway. (a) Phosphorylation of ERK1/2 and Elk-1 protein in control cultures of THP-1 cells and in cultures treated with the supernatants of scratch-injured human umbilical vein endothelial cells (HUVECs) for 5, 10, 15, 30, and 60 min. (b) Quantitative data for (a). (c) Phosphorylation of ERK1/2 and Elk-1 and expression of MMP-9 protein in control group, injury group, and PD98059 group. Control group was THP-1 cultures treated with the supernatants of HUVECs without scratch injury, while injury and PD98059 groups were cultures treated with supernatants of scratch-injured HUVECs for 6 hours. Cultures of the PD98059 group were pretreated with PD98095 for 1 h prior to HUVEC supernatants treatment. (d) Quantitative data for (c). ^*∗*^ *P* < 0.05 compared with control group; ^#^ *P* < 0.05 compared with rest groups; ^+^ *P* < 0.05 compared with injury group.

Next, we explored the roles of ERK1/2 and Elk-1 in MMP-9 expression. Phosphorylation of ERK1/2 and Elk-1 in THP-1 cells treated with the supernatants of scratch-injured HUVECs was detected by Western blot (Figures 5(a) and 5(b)). Phosphorylation of ERK1/2 and Elk-1 showed a similar pattern, both of which were gradually increased after induction. It peaked at 15 minutes and then decreased at 30 minutes but still remained significantly higher than normal levels. We then employed an inhibitor of ERK1/2 (PD98095) to attenuate the effect of ERK1/2. THP-1 cells were pretreated with PD98095 for 1 hour and then incubated with injured HUVECs supernatants. Western blot showed PD98095 not only dramatically suppressed ERK1/2 phosphorylation, but also significantly reduced Elk-1 phosphorylation and MMP-9 expression (Figures 5(c) and 5(d)). Together, our results suggest that scratch-injured endothelial supernatants induce MMP-9 expression in THP-1 cells, which could be mediated through ERK1/2 pathway with Elk-1, functioning downstream of ERK1/2.

#### 3.2.4. Telmisartan Reduces MMP-9 Expression by Inhibition of Elk-1 Activation in THP-1 Cells Stimulated by Scratch-Injured HUVECs Supernatants

**Figure 6:**
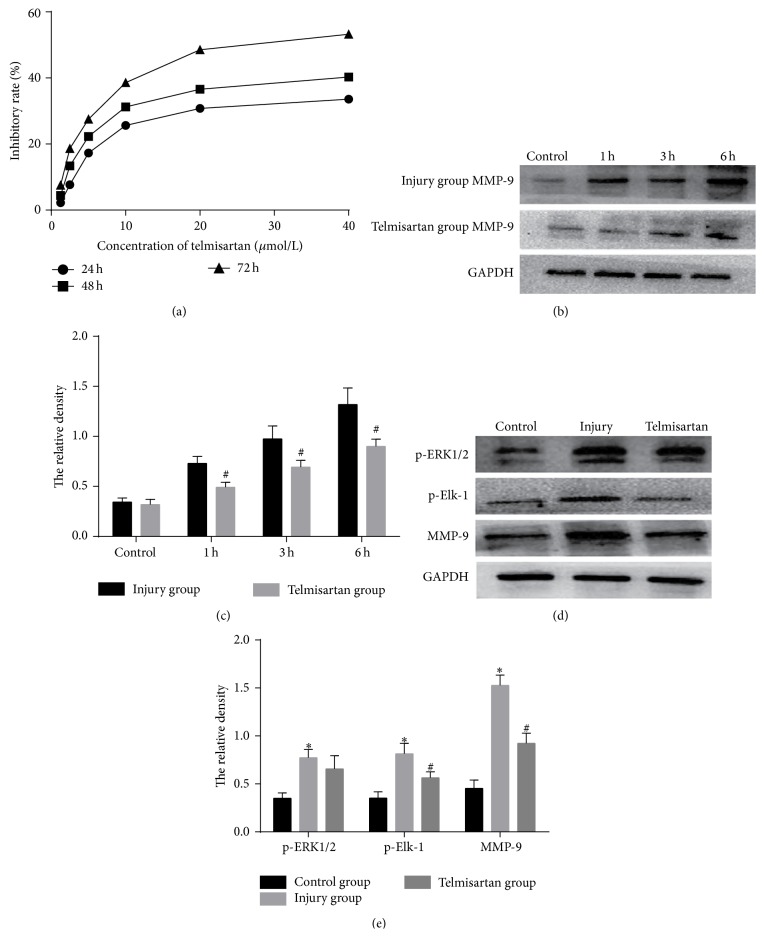
Telmisartan suppresses MMP-9 expression by inhibiting Elk-1 phosphorylation in THP-1 cells induced by scratch-injured human umbilical vein endothelial cells (HUVECs) supernatants. (a) Growth inhibiting effects of telmisartan on THP-1 cells. THP-1 cells were treated with 1.25, 2.5, 5, 10, 20, and 40 *µ*mol/L of telmisartan for 24, 48, and 72 h. Cell proliferation was determined by MTT assay. (b) MMP-9 protein expression in THP-1 cells incubated with scratch-injured HUVECs supernatants for 1, 3, and 6 h. THP-1 cells in telmisartan group were pretreated with telmisartan for 1 h before HUVECs supernatants treatment. (c) Quantitative data for (b). (d) ERK1/2 and Elk-1 phosphorylation and MMP-9 protein expression in control, injury, and telmisartan groups. Control group was THP-1 cultures treated with supernatants of HUVECs without injury, while injury group and telmisartan group were cultures treated with supernatants of HUVECs with scratch injury for 6 hours. Cultures of telmisartan group were pretreated with telmisartan for 1 h prior to HUVECs supernatants treatment. (e) Quantitative data for (d). Note: ^*∗*^ *P* < 0.05 compared with control group; ^#^ *P* < 0.05 compared with injury group.

Telmisartan was reported to reduce restenosis after stenting [28]. We aimed to investigate whether telmisartan inhibits MMP-9 expression via the ERK1/2/Elk-1 pathway. Firstly, we used an MTT assay to select the optimum concentration of telmisartan. The results showed that telmisartan inhibited cell growth in a dose- and time-dependent manner (Figure 6(a)). And low concentration of telmisartan (≤10 *µ*mol/L) affects the cell growth smaller. THP-1 cell growth approached a plateau above this 10 *µ*mol/L. Thus, we choose 10 *µ*mol/L as the concentrations of telmisartan for the next experiments.

Then we used Western blot to verify whether high expression of MMP-9 could be suppressed by telmisartan. The increase of MMP-9 expression in THP-1 cells treated with scratch-injured HUVEC supernatants for 1, 3, or 6 hours was all significantly inhibited by incubation with telmisartan before stimulation for 1 hour (Figures 6(b) and 6(c)). Afterwards, to assess whether ERK1/2 and Elk-1 are involved in the suppression of MMP-9 by telmisartan, ERK1/2 and Elk-1 phosphorylation and MMP-9 expression were measured in THP-1 cells pretreated with or without telmisartan for 6 hours. The results show that pretreatment with telmisartan efficiently downregulated the expression of p-Elk-1, but the reduction of p-ERK1/2 was not significantly different (Figures 6(d) and 6(e)). These results indicate that the inhibition effect of telmisartan on MMP-9 expression is via inhibition of Elk-1, but not of ERK1/2.

## 4. Discussion

Inflammation is well documented to play an important role in the pathophysiologic process after carotid and coronary artery stent placement, including the pathogenesis of ISR [4, 45, 46]. There is little research on the role of IL-1*β*, IL-6, and TGF-*β* after CAS. In the present study, serum levels of IL-1*β*, IL-6, and TGF-*β* are elevated immediately after CAS but remain elevated for less than 3 months. These results suggest that these cytokines may just take part in the acute process after CAS. IL-1*β* is a cytokine that plays a critical role in inflammatory complications after coronary stent placement, including ISR and stent thrombosis [10]. After it is released by injured cells, it stimulates endothelial cells to secrete chemokines and increases the expression of vascular adhesion molecules, which eventually leads to restenosis [10, 47, 48]. IL-6 works as a messenger cytokine in the expression of C-reactive protein, fibrinogen, and plasminogen activator inhibitor-1 and accelerates oxygen radical production [49, 50]. It is instantly released into circulating blood by mechanical plaque rupture [51, 52]. Furthermore, the level of IL-6 production is related to plaque instability. In patients undergoing CAS, the periprocedural levels of IL-6 were associated with new ischemic lesions and adverse clinical events [51–53]. TGF-*β* exerts its anti-inflammatory and anti-in-stent neointimal properties by suppressing adventitial T cell activation, hyaluronan deposition, cell proliferation, and adventitial matrix metalloproteinase-1 expression. This was confirmed in a pig model of coronary artery stenting [46], though the finding that TGF-*β* promotes neointimal blockage contradicts the findings in other pathologic conditions which indicate that blockade of TGF-*β* results in inhibition of neointimal formation [54, 55].

Previous studies confirmed that MMP-9 plays a critical role in the pathogenesis of ISR after coronary bare metal or drug-eluting stent placement, and MMP-9 was reported to be associated with coronary artery ISR in humans [6–9]. However, there has been far less attention on the relationship between MMP-9 expression and CAS [56]. In the present study, none of the patients were found to suffer from ISR in a 6-month follow-up time, while the normal rate of ISR after CAS is reported to range from 3% to 16.6% [57]. Some possible reasons for this inconsistency include small sample size, limited follow-up time, and a difference in patient selection in our study. However our study revealed that MMP-9 is associated with the pathophysiologic process after CAS for a long time as we found that circulating MMP-9 levels increased in both the acute and chronic phase in patients undergoing CAS. Our data is supported by a previous study by Hlawaty et al. who showed that MMP-9 activity was detectable after stenting in a rabbit model of ISR after CAS [56].

We developed and characterized a novel* in vitro* model of THP-1 cells activated by the supernatants of HUVECs scratched by pipette tips. Scratching the cell layer with a plastic pipette mimics the mechanical injury of stent implantation to endothelial cells, which is usually used in wound healing assays or in models to stimulate traumatic injury in astrocytes [58, 59]. In our model, injured HUVECs supernatants enhance THP-1 cells adhesion to HUVECs as well as migration and invasion, all of which are essential to monocytes in order to participate in the process of atherosclerosis and ISR, which demonstrates that scratch-injured HUVECs supernatants activate THP-1 cells. To our knowledge, ours is the first* in vitro* model that mimics the activation of monocytes stimulated by excretion of mechanically injured endothelial cells following stenting.

MCP-1 and VCAM-1 play important roles in monocytes activation, such as adhesion and recruitment of monocytes [41–44]. Moreover, MCP-1 has been shown to increase MMP-9 expression via ERK pathway [22]. Blood concentrations of MCP-1 and sVCAM-1 in patients who underwent stent implantation were elevated after stenting [38–40]. In our study, we measured the expression of MCP-1 and sVCAM-1 in the supernatants of scratch-injured HUVECs. We found MCP-1, but not sVCAM-1, in supernatants may contribute to the activation of THP-1 cells.

We used our new model to detect the molecular mechanisms of MMP-9 expression in monocytes after CAS. Several signaling pathways have been confirmed in the regulation of MMP-9 expression in different cell types, including ERK1/2, c-Jun N-terminal kinase (JNK), and p38 MAPKs [60, 61]. Accumulated data suggests that the ERK1/2 cascade is one of the main pathways that mediate MMP-9 expression and that the ERK1/2 inhibitor attenuates the expression of MMP-9 in various cells [62, 63]. After being activated by phosphorylation and translocated into nucleus, ERK1/2 catalyzes the phosphorylation of transcription factor Elk-1. Additionally, Elk-1 can be activated by JNK and p38 MAPKs as well [64]. Thus, Elk-1 may also play a critical role as a common downstream effector of various signaling cascades for MMP-9 expression. Elk-1 has been implicated as a regulator of ERK-mediated MMP-9 expression in several different pathologic conditions [24–26]. Hsieh et al. [24] reported that Elk-1 binds to an Ets-binding site located upstream of the transcription start site and takes part in the activation of the MMP-9 promoter in astrocytes. Consistent with these findings, our* in vitro* study showed that the inhibition of ERK1/2 attenuated the effect of increased Elk-1 phosphorylation and MMP-9 expression in monocytes stimulated by the supernatants of scratch-injured endothelial cells, suggesting that the ERK1/2/Elk-1 pathway mediates MMP-9 expression in monocytes after stent implantation.

Telmisartan has a unique agonistic effect on selective PPAR-*γ* in addition to its classic ARB effects. Though a meta-analysis revealed that use of ARBs cannot inhibit neointimal hyperplasia in patients after coronary stent implantation, the clinical trials that they analyzed did not use telmisartan [65]. Among ARBs, telmisartan has the most potent PPAR-*γ*-mediated effects, activating the receptor to 25%–30% of the maximum level achieved by full agonists such as pioglitazone and rosiglitazone. Telmisartan is confirmed to exert protective effects on vessels in several clinical trials in which patients underwent coronary artery stenting [27–29]. Compared with valsartan which has negligible PPAR-*γ* stimulating activity, telmisartan was associated with a significant decrease in late lumen loss, neointima volume, and inflammatory markers in two different prospective randomized studies [27, 29]. Additionally, in a recent retrospective study, telmisartan has been reported to reduce neointimal tissue proliferation with a lower ISR rate compared to enalapril [28]. Instead of inhibition of angiotensin II, these effects mainly occur due to the interaction between telmisartan and PPAR-*γ* ligand-binding domain, leading to a reduction in inflammatory conditions by suppressing the production of inflammatory factors [27, 29, 30, 66]. However, the molecular mechanism of telmisartan on MMP-9 expression has not been fully clarified. There are several reports showing that overexpression of MMP-9 could be downregulated by telmisartan in various pathological conditions [30–34], while losartan, a non-PPAR-*γ* agonistic ARB, does not decrease MMP-9 activity [33]. What is more, the decrease of MMP-9 after incubation with telmisartan can be inhibited by addition of PPAR-*γ* antagonist GW9662 [30, 33]. Rosiglitazone, a full agonist of PPAR-*γ*, was demonstrated to suppress the expression of MMP-9 by antagonizing the activities of the transcription factors such as AP-1, STAT, and NF-*κ*B [35, 36]. In the current study, telmisartan suppresses the expression of MMP-9 in monocytes induced by supernatants of scratch-injured endothelial cells by inhibition of Elk-1 activation, but not ERK1/2. Elk-1-target gene* FOS* encoded transcription factor c-Fos, a component of AP-1, and AP-1 can upregulate the expression of MMP-9 [64, 67, 68]. Additionally, Yang et al. reported that activation of ERK2 is required for inducing PPAR-*γ* expression [69]. Thus, our* in vitro* study provides a mechanistic basis for how telmisartan regulates MMP-9 expression in activated monocytes.

In conclusion, our extensive study shows that the inflammatory cytokines, IL-1*β*, IL-6, TGF-*β*, and MMP-9, participate in the early inflammatory process after CAS. MMP-9 also takes part in the chronic phase after stent implantation. Furthermore, we developed and evaluated a new* in vitro* model of THP-1 cells activated by the supernatants of scratch-injured HUVECs to mimic monocytes activated by excretion of endothelial cells mechanically injured by CAS. In this model, MMP-9 expression can be regulated by suppression of ERK1/2/Elk-1 pathway. We also demonstrated that the protective effects of telmisartan after stenting may be partly because of its inhibitory effects on MMP-9 expression by suppressing Elk-1, but not ERK1/2, in activated monocytes after stenting. These findings provide insights and options for future clinical interventions to suppress complications of CAS.

## Figures and Tables

**Table 1:** Baseline characteristics of patients scheduled for CAS.

Parameter	*n* = 74	%
Age, years	66.15 ± 7.39	
Sex, male	48	64.86
Hypertension	51	68.92
Coronary artery disease	9	12.16
Diabetes	23	31.08
Impaired glucose tolerance	14	18.92
Hyperlipidemia	54	72.97
Hyperthyroidism	1	1.35
Symptomatic stenosis	43	58.11
Stenosis degree		
50%~70%	15	20.27
>70%	59	79.73

Values are given as mean ± SD or *n* (%).

## Nonstandard Abbreviations and Acronyms

ARB: Angiotensin II receptor blocker
CAS: Carotid artery stenting
ERK1/2: Extracellular signal-regulated kinases 1/2
IL-1*β*: Interleukin-1*β*
IL-6: Interleukin-6
ISR: In-stent restenosis
MCP-1: Monocyte chemoattractant protein-1
MMP-9: Matrix metalloproteinase-9
PPAR-*γ*: Peroxisome proliferator-activated receptor-gamma
sVCAM-1: Soluble vascular cell adhesion molecule-1
TGF-*β*: Transforming growth factor-*β*
VCAM-1: Vascular cell adhesion molecule-1.

## References

[1] Lovett J. K., Coull A. J., Rothwell P. M. (2004). Early risk of recurrence by subtype of ischemic stroke in population-based incidence studies. *Neurology*.

[2] Wasser K., Schnaudigel S., Wohlfahrt J., Psychogios M.-N., Knauth M., Gröschel K. (2011). Inflammation and in-stent restenosis: the role of serum markers and stent characteristics in carotid artery stenting. *PLoS ONE*.

[3] Inoue T., Croce K., Morooka T., Sakuma M., Node K., Simon D. I. (2011). Vascular inflammation and repair: implications for re-endothelialization, restenosis, and stent thrombosis. *JACC: Cardiovascular Interventions*.

[4] Khouzam R. N., Shaheen M., Aziz R. K., Ibebuogu U. N. (2012). The important role of inflammatory biomarkers pre and post bare-metal and drug-eluting stent implantation. *Canadian Journal of Cardiology*.

[5] Welt F. G. P., Rogers C. (2002). Inflammation and restenosis in the stent era. *Arteriosclerosis, Thrombosis, and Vascular Biology*.

[6] Jones G. T., Tarr G. P., Phillips L. V., Wilkins G. T., van Rij A. M., Williams M. J. A. (2009). Active matrix metalloproteinases 3 and 9 are independently associated with coronary artery in-stent restenosis. *Atherosclerosis*.

[7] Liu W., Liu Y., Jiang H. (2013). Plasma levels of interleukin 18, interleukin 10, and matrix metalloproteinase-9 and -137G/C polymorphism of interleukin 18 are associated with incidence of in-stent restenosis after percutaneous coronary intervention. *Inflammation*.

[8] Katsaros K. M., Kastl S. P., Zorn G. (2010). Increased restenosis rate after implantation of drug-eluting stents in patients with elevated serum activity of matrix metalloproteinase-2 and -9. *JACC: Cardiovascular Interventions*.

[9] Jones G. T., Kay I. P., Chu J. W. S. (2006). Elevated plasma active matrix metalloproteinase-9 level is associated with coronary artery in-stent restenosis. *Arteriosclerosis, Thrombosis, and Vascular Biology*.

[10] Sardella G., Mariani P., D'Alessandro M. (2006). Early elevation of interleukin-1*β* and interleukin-6 levels after bare or drug-eluting stent implantation in patients with stable angina. *Thrombosis Research*.

[11] Joner M., Farb A., Cheng Q. (2007). Pioglitazone inhibits in-stent restenosis in atherosclerotic rabbits by targeting transforming growth factor-*β* and MCP-1. *Arteriosclerosis, Thrombosis, and Vascular Biology*.

[12] Chen Q., Jin M., Yang F., Zhu J., Xiao Q., Zhang L. (2013). Matrix metalloproteinases: inflammatory regulators of cell behaviors in vascular formation and remodeling. *Mediators of Inflammation*.

[13] Zalba G., Fortuño A., Orbe J. (2007). Phagocytic NADPH oxidase-dependent superoxide production stimulates matrix metalloproteinase-9: implications for human atherosclerosis. *Arteriosclerosis, Thrombosis, and Vascular Biology*.

[14] Feldman L. J., Mazighi M., Scheuble A. (2001). Differential expression of matrix metalloproteinases after stent implantation and balloon angioplasty in the hypercholesterolemic rabbit. *Circulation*.

[15] Welt F. G. P., Tso C., Edelman E. R. (2003). Leukocyte recruitment and expression of chemokines following different forms of vascular injury. *Vascular Medicine*.

[16] Liu Y., Imanishi T., Ikejima H. (2010). Association between circulating monocyte subsets and in-stent restenosis after coronary stent implantation in patients with ST-elevation myocardial infarction. *Circulation Journal*.

[17] Virmani R., Farb A. (1999). Pathology of in-stent restenosis. *Current Opinion in Lipidology*.

[18] Amorino G. P., Hoover R. L. (1998). Interactions of monocytic cells with human endothelial cells stimulate monocytic metalloproteinase production. *American Journal of Pathology*.

[19] Bobryshev Y. V. (2006). Monocyte recruitment and foam cell formation in atherosclerosis. *Micron*.

[20] Yamamoto Y., Osanai T., Nishizaki F. (2012). Matrix metalloprotein-9 activation under cell-to-cell interaction between endothelial cells and monocytes: possible role of hypoxia and tumor necrosis factor-*α*. *Heart and Vessels*.

[21] Langlois A., Chouinard F., Flamand N., Ferland C., Rola-Pleszczynski M., Laviolette M. (2009). Crucial implication of protein kinase C (PKC)-*δ*, PKC-*ζ*, ERK-1/2, and p38 MAPK in migration of human asthmatic eosinophils. *Journal of Leukocyte Biology*.

[22] Tang C.-H., Tsai C.-C. (2012). CCL2 increases MMP-9 expression and cell motility in human chondrosarcoma cells via the Ras/Raf/MEK/ERK/NF-*κ*B signaling pathway. *Biochemical Pharmacology*.

[23] Roskoski R. (2012). ERK1/2 MAP kinases: structure, function, and regulation. *Pharmacological Research*.

[24] Hsieh H.-L., Wu C.-Y., Yang C.-M. (2008). Bradykinin induces matrix metalloproteinase-9 expression and cell migration through a PKC-*δ*-dependent ERK/Elk-1 pathway in astrocytes. *Glia*.

[25] Choi B. D., Jeong S. J., Wang G. (2011). Secretory leukocyte protease inhibitor is associated with MMP-2 and MMP-9 to promote migration and invasion in SNU638 gastric cancer cells. *International Journal of Molecular Medicine*.

[26] Ahmad N., Wang W., Nair R., Kapila S. (2012). Relaxin induces matrix-metalloproteinases-9 and -13 via RXFP1: induction of MMP-9 involves the PI3K, ERK, Akt and PKC-*ζ* pathways. *Molecular and Cellular Endocrinology*.

[27] Hong S. J., Choi S. C., Ahn C. M., Park J. H., Kim J. S., Lim D.-S. (2011). Telmisartan reduces neointima volume and pulse wave velocity 8 months after zotarolimus-eluting stent implantation in hypertensive type 2 diabetic patients. *Heart*.

[28] Yokoyama J., Higuma T., Tomita H. (2009). Impact of telmisartan on coronary stenting in patients with acute myocardial infarction compared with enalapril. *International Journal of Cardiology*.

[29] Hong S. J., Shim W. J., Choi J. I. (2007). Comparison of effects of telmisartan and valsartan on late lumen loss and inflammatory markers after sirolimus-eluting stent implantation in hypertensive patients. *American Journal of Cardiology*.

[30] Zhu C., Zhang X., Qiao H. (2012). The intrinsic PEDF is regulated by PPAR*γ* in permanent focal cerebral ischemia of rat. *Neurochemical Research*.

[31] Nakamura T., Inoue T., Suzuki T. (2008). Comparison of renal and vascular protective effects between telmisartan and amlodipine in hypertensive patients with chronic kidney disease with mild renal insufficiency. *Hypertension Research*.

[32] Araújo A. A., Souza T. O., Moura L. M. (2013). Effect of telmisartan on levels of IL-1, TNF-*α*, down-regulated COX-2, MMP-2, MMP-9 and RANKL/RANK in an experimental periodontitis model. *Journal of Clinical Periodontology*.

[33] Maejima Y., Okada H., Haraguchi G. (2011). Telmisartan, a unique ARB, improves left ventricular remodeling of infarcted heart by activating PPAR gamma. *Laboratory Investigation*.

[34] Yokota T., Osanai T., Hanada K. (2010). Effects of telmisartan on markers of ventricular remodeling in patients with acute myocardial infarction: comparison with enalapril. *Heart and Vessels*.

[35] Ricote M., Li A. C., Willson T. M., Kelly C. J., Glass C. K. (1998). The peroxisome proliferator-activated receptor-*γ* is a negative regulator of macrophage activation. *Nature*.

[36] Herzlich A. A., Tuo J., Chan C.-C. (2008). Peroxisome proliferator-activated receptor and age-related macular degeneration. *PPAR Research*.

[37] Müller I., Endo T., Thiel G. (2010). Regulation of AP-1 activity in glucose-stimulated insulinoma cells. *Journal of Cellular Biochemistry*.

[38] Hong S. J., Kim S. T., Kim T.-J. (2010). Cellular and molecular changes associated with inhibitory effect of pioglitazone on neointimal growth in patients with type 2 diabetes after zotarolimus-eluting stent implantation. *Arteriosclerosis, Thrombosis, and Vascular Biology*.

[39] Munk P. S., Breland U. M., Aukrust P., Skadberg O., Ueland T., Larsen A. I. (2011). Inflammatory response to percutaneous coronary intervention in stable coronary artery disease. *Journal of Thrombosis and Thrombolysis*.

[40] Patti G., Chello M., Pasceri V. (2005). Dexamethasone-eluting stents and plasma concentrations of adhesion molecules in patients with unstable coronary syndromes: results of the historically controlled SESAME study. *Clinical Therapeutics*.

[41] Miller M. D., Krangel M. S. (1992). Biology and biochemistry of the chemokines: a family of chemotactic and inflammatory cytokines. *Critical Reviews in Immunology*.

[42] van den Berg R. H., Faber-Krol M. C., Sim R. B., Dana M. R. (1998). The first subcomponent of complement, C1q, triggers the production of IL-8, IL-6, and monocyte chemoattractant peptide-1 by human umbilical vein endothelial cells. *The Journal of Immunology*.

[43] Chuang S.-Y., Yang S.-H., Pang J.-H. S. (2011). Cilostazol reduces MCP-1-induced chemotaxis and adhesion of THP-1 monocytes by inhibiting CCR2 gene expression. *Biochemical and Biophysical Research Communications*.

[44] Dansky H. M., Barlow C. B., Lominska C. (2001). Adhesion of monocytes to arterial endothelium and initiation of atherosclerosis are critically dependent on vascular cell adhesion molecule-1 gene dosage. *Arteriosclerosis, Thrombosis, and Vascular Biology*.

[45] Setacci C., de Donato G., Chisci E. (2008). Deferred urgency carotid artery stenting in symptomatic patients: clinical lessons and biomarker patterns from a prospective registry. *European Journal of Vascular and Endovascular Surgery*.

[46] Chung I.-M., Kim J., Pak Y. K. (2010). Blockade of TGF-*β* by catheter-based local intravascular gene delivery does not alter the in-stent neointimal response, but enhances inflammation in pig coronary arteries. *International Journal of Cardiology*.

[47] Voisard R., Munder U., von Muller L., Baur R., Hombach V. (2011). Direct inhibitory effects of Ganciclovir on ICAM-1 expression and proliferation in human coronary vascular cells (SI/MPL-ratio: >1). *Medical Science Monitor*.

[48] Dinarello C. A. (1998). Interleukin-1, interleukin-1 receptors and interleukin-1 receptor antagonist. *International Reviews of Immunology*.

[49] Packard R. R. S., Libby P. (2008). Inflammation in atherosclerosis: from vascular biology to biomarker discovery and risk prediction. *Clinical Chemistry*.

[50] Kofler S., Nickel T., Weis M. (2005). Role of cytokines in cardiovascular diseases: a focus on endothelial responses to inflammation. *Clinical Science*.

[51] Abe Y., Sakaguchi M., Furukado S. (2010). Interleukin-6 release after carotid artery stenting and periprocedural new ischemic lesions. *Journal of Cerebral Blood Flow and Metabolism*.

[52] Abe Y., Sakaguchi M., Furukado S. (2010). Associations of local release of inflammatory biomarkers during carotid artery stenting with plaque echogenicity and calcification. *Cerebrovascular Diseases*.

[53] Versaci F., Reimers B., Prati F. (2012). Prediction of cardiovascular events by inflammatory markers in patients undergoing carotid stenting. *Mayo Clinic Proceedings*.

[54] Yamamoto K., Morishita R., Tomita N. (2000). Ribozyme oligonucleotides against transforming growth factor-*β* inhibited neointimal formation after vascular injury in rat model: potential application of ribozyme strategy to treat cardiovascular disease. *Circulation*.

[55] Lutgens E., Gijbels M., Smook M. (2002). Transforming growth factor-*β* mediates balance between inflammation and fibrosis during plaque progression. *Arteriosclerosis, Thrombosis, and Vascular Biology*.

[56] Hlawaty H., Jacob M.-P., Louedec L. (2009). Leukotriene receptor antagonism and the prevention of extracellular matrix degradation during atherosclerosis and in-stent stenosis. *Arteriosclerosis, Thrombosis, and Vascular Biology*.

[57] Lal B. K., Beach K. W., Roubin G. S. (2012). Restenosis after carotid artery stenting and endarterectomy: a secondary analysis of CREST, a randomised controlled trial. *The Lancet Neurology*.

[58] Faber-Elman A., Solomon A., Abraham J. A., Marikovsky M., Schwartz M. (1996). Involvement of wound-associated factors in rat brain astrocyte migratory response to axonal injury: in vitro simulation. *Journal of Clinical Investigation*.

[59] Gao K., Wang C. R., Jiang F. (2013). Traumatic scratch injury in astrocytes triggers calcium influx to activate the JNK/c-Jun/AP-1 pathway and switch on GFAP expression. *Glia*.

[60] Gomes L. R., Terra L. F., Wailemann R. A. M., Labriola L., Sogayar M. C. (2012). TGF-*β*1 modulates the homeostasis between MMPs and MMP inhibitors through p38 MAPK and ERK1/2 in highly invasive breast cancer cells. *BMC Cancer*.

[61] Yao J., Xiong S., Klos K. (2001). Multiple signaling pathways involved in activation of matrix metalloproteinase-9 (MMP-9) by heregulin-*β*1 in human breast cancer cells. *Oncogene*.

[62] Mohammad G., Siddiquei M. M., Nawaz M. I., Abu El-Asrar A. M. (2013). The ERK1/2 inhibitor U0126 attenuates diabetes-induced upregulation of MMP-9 and biomarkers of inflammation in the retina. *Journal of Diabetes Research*.

[63] Lee E.-J., Park S.-S., Kim W.-J., Moon S.-K. (2012). IL-5-induced migration via ERK1/2-mediated MMP-9 expression by inducing activation of NF-*κ*B in HT1376 cells. *Oncology Reports*.

[64] Kasza A. (2013). Signal-dependent Elk-1 target genes involved in transcript processing and cell migration. *Biochimica et Biophysica Acta*.

[65] Kun X., Yong L., Bo J., Hai-Ming S. (2011). Neointimal hyperplasia inhibition effect of angiotensin II type 1 receptor blockers in patients after coronary stent implantation: a meta-analysis. *American Journal of Cardiovascular Drugs*.

[66] Jiang C., Ting A. T., Seed B. (1998). PPAR-*γ* agonists inhibit production of monocyte inflammatory cytokines. *Nature*.

[67] Ozanne B. W., McGarry L., Spence H. J. (2000). Transcriptional regulation of cell invasion: AP-1 regulation of a multigenic invasion programme. *European Journal of Cancer*.

[68] Ozanne B. W., Spence H. J., McGarry L. C., Hennigan R. F. (2007). Transcription factors control invasion: AP-1 the first among equals. *Oncogene*.

[69] Yang S.-L., Chen S.-L., Wu J.-Y., Ho T.-C., Tsao Y.-P. (2010). Pigment epithelium-derived factor induces interleukin-10 expression in human macrophages by induction of PPAR gamma. *Life Sciences*.

